# 14-3-3η Promotes Invadosome Formation via the FOXO3–Snail Axis in Rheumatoid Arthritis Fibroblast-like Synoviocytes

**DOI:** 10.3390/ijms23010123

**Published:** 2021-12-23

**Authors:** Maleck Kadiri, Martine Charbonneau, Catherine Lalanne, Kelly Harper, Frédéric Balg, Anthony Marotta, Claire M. Dubois

**Affiliations:** 1 Department of Immunology and Cell Biology, Université de Sherbrooke, Sherbrooke, QC J1H 5N4, Canada; maleck.Kadiri@usherbrooke.ca (M.K.); martine.charbonneau@usherbrooke.ca (M.C.); catlal1113@gmail.com (C.L.); kelly.harper@usherbrooke.ca (K.H.); 2 Department of Orthopedic Surgery, Université de Sherbrooke, Sherbrooke, QC J1H 5N4, Canada; Frederic.Balg@USherbrooke.ca; 3 Augurex Life Sciences Corp., Vancouver, BC V5T 4T5, Canada; dramarotta@gmail.com

**Keywords:** 14-3-3η, FOXO3, Snail, rheumatoid arthritis, fibroblast-like synoviocytes, invadosome

## Abstract

Erosive destruction of joint structures is a critical event in the progression of rheumatoid arthritis (RA), in which fibroblast-like synoviocytes (FLS) are the primary effectors. We previously reported that the ability of RA FLS to degrade extracellular matrix (ECM) components depends on the formation of actin-rich membrane protrusions, called invadosomes, through processes that remain elusive. 14-3-3η belongs to a family of scaffolding proteins involved in a wide range of cellular functions, and its expression is closely related to joint damage and disease activity in RA patients. In this study, we sought to assess the role of 14-3-3η in joint damage by examining its contribution to the invadosome formation phenotype of FLS. Using human primary FLS, we show that 14-3-3η expression is closely associated with their ability to form invadosomes. Furthermore, knockdown of 14-3-3η using shRNAs decreases the level of invadosome formation in RA FLS, whereas addition of the recombinant protein to FLS from healthy individuals promotes their formation. Mechanistic studies suggest that 14-3-3η regulates invadosome formation by increasing Snail expression, a mechanism that involves nuclear exclusion of the transcription repressor FOXO3. Our results implicate the 14-3-3η–FOXO3–Snail axis in promoting the aggressive ECM-degrading phenotype of RA FLS, and suggest a role for this scaffolding protein in cartilage degradation.

## 1. Introduction

Rheumatoid arthritis (RA) is a common autoimmune disease characterized by chronic inflammation with progressive destruction of articular cartilage and bone, resulting in pain, functional disability, and premature death. Articular cartilage is considered as a main target tissue in RA, which becomes damaged as a result of the formation of an aggressive tumor-like structure composed of a hyperplasic synovial membrane called pannus [1]. Today, the diagnosis of RA is based on the 2010 classification criteria for RA [2], which emphasize early patient identification and prompt initiation of treatments to minimize irreversible joint destruction and decreased quality of life for patients [3]. Recently, 14-3-3η has emerged as a new biomarker of RA that may improve its early diagnosis [4,5,6,7]. 14-3-3η belongs to a family of ubiquitously expressed intracellular scaffolding proteins. There are seven highly conserved isoforms—β, ζ, γ, σ, ε, θ, and η—which are known to bind to a variety of phosphoserine/threonine proteins involved in the regulation of various cellular processes including cell cycle, apoptosis, signal transduction, transcription regulation, adhesion, and invasion [8,9,10]. Among the different isoforms, 14-3-3η is the one mainly detected in the serum and synovial fluid of RA patients [11]. Of note, the levels of 14-3-3η detected were up to fivefold higher in synovial fluid than in the corresponding serum, suggesting that hyperplasic synovium is likely the source of this protein [12]. Furthermore, serum levels of 14-3-3η were significantly higher in patients with radiographic evidence of joint damage, and the addition of 14-3-3η to inflammatory cells promoted the induction of inflammatory transcripts such as metalloproteinase (MMP)-1 and MMP-3. These data highlight a potential link between 14-3-3η and joint destruction in RA patients [4], but important questions remain as to the mechanism(s) by which 14-3-3η might participate in the processes leading to joint destruction

As one of the major components of the hyperplasic RA synovial membrane, fibroblast-like synoviocytes (FLS) play a key role in the pathological processes leading to joint damage. RA FLS resemble immature, transformed fibroblasts with a high proliferation rate and in vitro and in vivo invasive capacity [13,14]. A direct consequence of RA FLS transformation is their increased ability to degrade and invade cartilage. In fact, these cells exhibit a unique aggressive tumor-like behavior, known to actively drive persistent degradation of adjacent articular cartilage and to promote erosion of subchondral bone via the production of several mediators such as MMPs, A Disintegrin And Metalloproteinase with Thrombospondin Motif (ADAMTS)-4 and ADAMTS-5, cathepsins, proinflammatory cytokines, and bone resorption mediators [15,16]. Interestingly, recent findings demonstrate that degradation of articular cartilage is dependent on the formation of actin-rich membrane protrusions: the invadosomes [17]. These extracellular matrix degradation devices were originally described in tumor cells, associated with systemic dissemination and metastasis, and are enriched in MMPs [18]. In RA, the invadosomal structures have been shown to contain invadosome markers such as actin components, Src signaling molecules, and high levels of ECM-degrading metalloproteinases, and to be strategically located at the cartilage–synovial membrane interface [17]. Further studies have identified some of the molecular actors involved in their production, such as an autocrine activation loop that involves TGF-β, PDGF receptor, and Snail [19,20]. Importantly, interference with invadosome formation in RA FLS strongly inhibited matrix degradation in vitro, as well as cartilage degradation, in a rat model of arthritis [17,21]. These observations suggested that invadosomes are directly involved in the process of joint degradation. However, the molecular mechanisms that regulate invadosome formation by RA FLS remain to be fully understood.

FLS transformation is a critical event in the acquisition of an aggressive and invasive phenotype [15,22]. In recent years, it has been demonstrated that downstream signaling pathways and transcription factors (TFs) activated by pro-inflammatory mediators are required for this transformation [23]. In particular, dysregulation of the activity of TFs initially involved in embryonic development and cancer, such as SOX, Snail, and FOXO, has been reported [24,25,26,27]. Among them, FOXO family members have been recently shown to be critically linked to chronic inflammatory diseases, such as arthritis [28]. FOXO proteins are a subgroup of the Forkhead family of transcription factors, which includes three ubiquitously expressed genes, *FOXO1*,*3*,*4*, as well as *FOXO6*, the expression of which is more restricted to the brain and liver [29]. Several studies have associated the downregulation or inactivation of FOXOs with reduced apoptosis and enhanced migration and invasion of different cancer cell lines, consistent with their role as tumor suppressors [30,31,32]. In RA, the loss of FOXO3 function was associated with reduced inflammation and joint damage [33,34,35]. Downregulation of FOXO1 expression was also required to promote the survival of RA FLS [26], and inactivation of FOXO3 was shown to be an important event for FLS-mediated inflammation [35]. However, the exact mechanism by which FOXOs were regulated in RA remained unknown. Intriguingly, previous research has revealed that the interaction of 14-3-3 proteins with FOXO family members is an important step in regulating their functions. 14-3-3ε binding to FOXO1 was shown to block its DNA binding and accelerate its nuclear export, thereby impairing the expression of genes involved in cell death [36,37]. Another member of the 14-3-3 family (14-3-3ζ) was shown to enhance the migration of tongue squamous cell carcinoma cells through the relocalization of FOXO3 from the nucleus to the cytoplasm [38]. Taken together, these findings suggest a potential mechanism by which 14-3-3η may be associated with FLS-induced cartilage destruction.

Here, we sought to better understand the role of 14-3-3η in joint damage by examining its potential contribution to the aggressive invadosome-forming phenotype of RA FLS and to identify the mechanism involved. We show that 14-3-3η expression is closely associated with the invadosome-forming phenotype in RA FLS. Knockdown of 14-3-3η decreases the level of invadosome formation in RA FLS, whereas addition of the recombinant protein to H FLS promotes their formation. Furthermore, our results suggest that 14-3-3η regulates invadosome formation by increasing Snail expression, a mechanism that involves nuclear exclusion of FOXO3. These results highlight an important role for 14-3-3η in invadosome formation and provide a novel mechanism that implicates the 14-3-3η–FOXO3–Snail axis in promoting the aggressive ECM-degrading phenotype of FLS in RA.

## 2. Results

### 2.1. Increased Expression of 14-3-3η in FLS and Synovial Tissue Sections from RA Patients Correlates with Invadosome Formation

**Figure 1:**
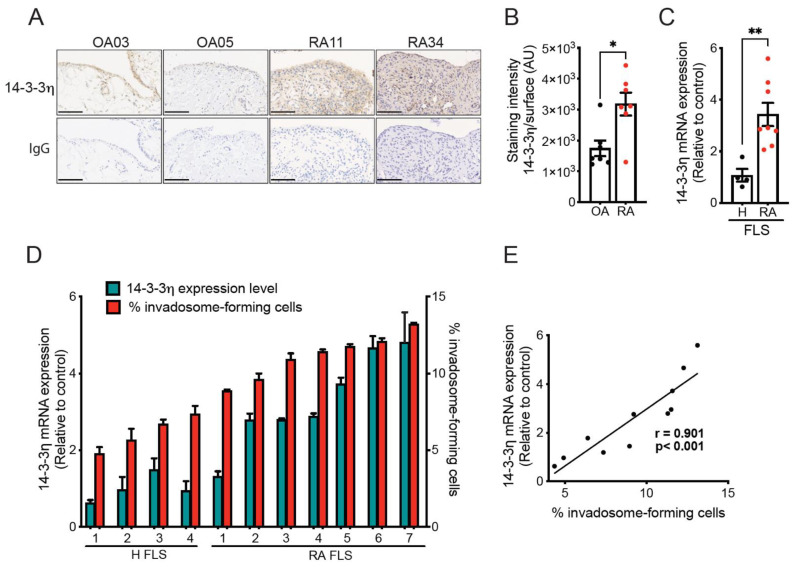
14-3-3η expression in synovial tissue sections and primary cultures of H FLS and RA FLS. (**A**) Representative images (original magnification 40X) of 14-3-3η expression in paraffin-embedded synovial tissue sections from RA and OA patients (upper panel). Isotype matched control antibody (IgG) staining is presented in the lower panels. Scale bar = 100 µm. (**B**) The associated graph represents the intensity of labeling in arbitrary units (AU) for 7 OA and 8 RA patients. (**C**) RT-qPCR analysis of 14-3-3η mRNA expression in H FLS (from 4 healthy individuals) and RA FLS (from 7 RA patients) using RPLP0 as a reference gene. The results are expressed as mean mRNA expression relative to the control. (**D**) Relationship between 14-3-3η mRNA expression and invadosome formation in H FLS and RA FLS. H FLS (from 4 healthy individuals) and RA FLS (from 7 RA patients) were cultured on Oregon Green^488^-conjugated gelatin for 48 h, and the percentage of cells forming invadosomes was counted in 3 fields of 100 cells (*n* = 2–4). mRNA expression was also analyzed via qPCR for matched cell lines. (**E**) The correlation between the 14-3-3η mRNA level and invadosome formation was evaluated using the Pearson’s correlation coefficient. Data represent the mean ± SEM * *p* < 0.05 and ** *p* < 0.01, unpaired *t*-test.

To investigate the potential role of 14-3-3η in joint degradation, we first determined the expression level of 14-3-3η in synovial tissue samples from RA and OA patients collected during open surgery. Immunohistochemistry analysis of tissue sections showed enhanced staining of 14-3-3η in RA synovial tissues compared to that in OA tissues, with the more intense signal distributed primarily in the hyperplasic synovial intimal lining (Figure 1A). Quantification of the staining intensities confirmed the increased expression of 14-3-3η in RA tissues (Figure 1B). In addition, gene expression analysis of 14-3-3η in primary cultures of fibroblast-like synoviocytes (FLS) indicated that 14-3-3η mRNA levels were significantly increased in FLS from RA patients when compared to synoviocytes from healthy individuals (H FLS) (Figure 1C).

ECM degradation and subsequent joint damage was previously shown to be mediated by invadosome structures formed by RA FLS [19,20]. To determine the possible link between 14-3-3η expression and joint destruction, we compared the ability of FLS to produce invadosomes with the level of 14-3-3η mRNA expression using FLS from different RA patients and healthy individuals. The results showed that 14-3-3η gene expression was associated with the level of invadosomes formed by the different H FLS or RA FLS (Figure 1D). Further analysis indicated a strong positive correlation between 14-3-3η mRNA level and invadosome formation (*r* = 0.901; *p* < 0.001) (Figure 1E). These results indicate that 14-3-3η expression is closely associated with the aggressive, invadosome-forming phenotype in FLS.

### 2.2. 14-3-3η Is a Regulator of Invadosome Formation in FLS

**Figure 2:**
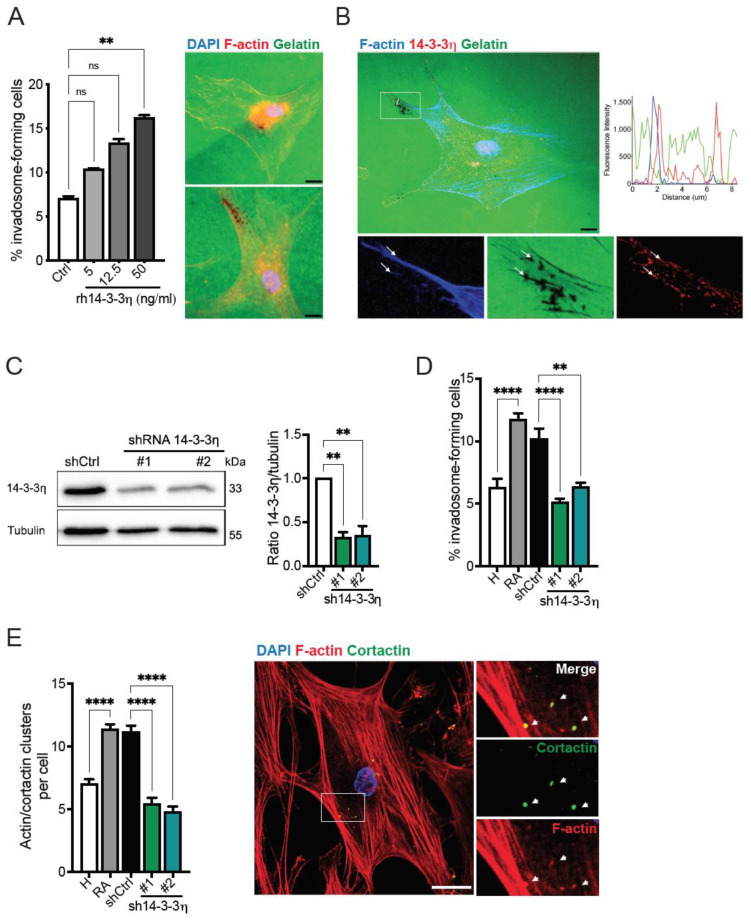
Regulation of invadosome formation by 14-3-3η. (**A**) H FLS were plated on Oregon green^488^-conjugated gelatin coverslips for 48 h in the presence or absence of human recombinant 14-3-3η protein (rh14-3-3η) used at the indicated concentrations (*n* = 4). The associated micrographs show representative invadosome formation (stained for F-actin (red), gelatin (green), and nuclei (DAPI, blue)) of untreated (ctrl) and rh14-3-3η (50 ng/mL) treated cells. Scale bar = 20 µm. (**B**) Representative confocal microscopy images and corresponding zoom of the boxed area of RA FLS cultured on Oregon green^488^ gelatin for 48 h and stained for F-actin (blue), nuclei (DAPI, blue), 14-3-3η (red), and gelatin (green). The boxed area represents a higher magnification of invadosome structures. Arrows represent F-actin and 14-3-3η colocalization at areas of gelatin degradation (loss of green fluorescence). The associated graph shows the fluorescence intensity profile of F-actin, 14-3-3η, and gelatin through the plane indicated by the solid white line of the merged high magnification. Original magnification, 60X. Scale bar = 100 µm. (**C**–**E**) RA FLS were transduced with non-targeting control (shctrl) or 14-3-3η-targeting shRNAs (#1 and #2). (**C**) Western blot analysis of 14-3-3η expression. The associated graph represents the quantification of 3 independent experiments. (**D**) H FLS or RA FLS were cultured on Oregon green^488^-conjugated gelatin coverslips for 48 h, and the percentage of invadosome-forming cells was calculated (*n* = 4–9). (E) H FLS or RA FLS were cultured on non-fluorescent gelatin for 6 h, and the number of actin–cortactin clusters was counted for 25 cells per condition (*n* = 4). A representative image of actin–cortactin clusters in RA FLS stained for F-actin (red), cortactin (green), and nuclei (DAPI, blue) is shown. The boxed area represents a higher magnification of actin-cortactin clusters. Arrows represent F-actin and cortactin puncta. Scale bar = 20 µm. Data represent the mean ± SEM. ** *p* < 0.01 and **** *p* < 0.0001, ns = non-significant, one-way ANOVA.

To determine whether 14-3-3η is involved in invadosome formation, we first evaluated the ability of H FLS to form invadosomes in the presence or absence of human recombinant 14-3-3η protein (rh14-3-3η). The addition of rh14-3-3η increased the level of invadosome formation in a concentration-dependent manner, with a significant augmentation observed at a concentration of 50 ng/mL (Figure 2A). To further characterize the implication of 14-3-3η in invadosome formation, RA FLS were stained for 14-3-3η and F-actin, and cellular expression of 14-3-3η was analyzed by confocal microscopy. As expected [39], the results indicated the presence of 14-3-3η in both cytoplasmic and nuclear compartments of RA FLS. We also found 14-3-3η localization at actin-rich and ECM-degradation areas characteristic of invadosomes (Figure 2B). We next reduced 14-3-3η gene expression in RA FLS using shRNAs that target two independent regions of the mRNA. The efficacy of the shRNAs was validated by Western blot analysis (Figure 2C). The results indicated that the knockdown of 14-3-3η markedly diminished the percentage of cells forming invadosomes (Figure 2D). Similarly, the number of invadosome core structures identified by cortactin and actin, two markers of invadosomes, was significantly reduced in 14-3-3η-depleted cells (Figure 2E). These results indicate that 14-3-3η is an important mediator of invadosome formation in RA FLS.

### 2.3. 14-3-3η Regulates Snail Expression and Activity

**Figure 3:**
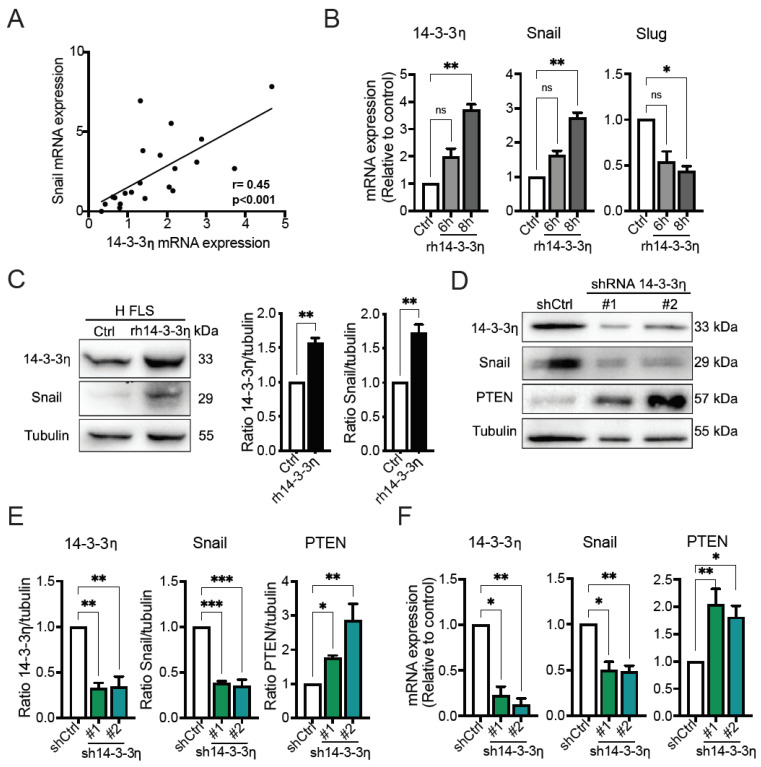
The role of 14-3-3η in regulating Snail expression and activity in RA FLS. (**A**) The correlation between 14-3-3η and Snail mRNA expression was measured via the Pearson’s correlation coefficient using data from different primary cultures of H FLS (*n* = 8) and RA FLS (*n* = 13). (**B**) H FLS were serum starved and treated with 50 ng/mL of rh14-3-3η for 6 or 8 h for RT-qPCR or (**C**) 48 h for Western blotting. Cell lysates were analyzed for the expression levels of 14-3-3η and Snail (n ≥ 3). Quantification of the Western blot data is presented in the right panel. (**D**–**F**) RA FLS were transduced with control or 14-3-3η targeting shRNAs. (**D**,**E**) Western blot and (**F**) RT-qPCR for the expression of 14-3-3η, Snail, and PTEN were performed. The associated graphs represent the quantification of the data (*n* = 3–5). Data represent the mean ± SEM, *n* ≥ 3. * *p* < 0.05, ** *p* < 0.01, and *** *p* < 0.001, ns = non-significant, one-way ANOVA or unpaired *t* test.

We previously reported that the transcription factor Snail is required for the increased capacity of RA FLS to form invadosomes compared to H FLS [19]. It has also been reported that 14-3-3 proteins are implicated in Snail expression in lung and glioma cancer cell lines [40,41]. To investigate the possibility that 14-3-3η is involved in Snail expression in our cell model, we first measured the degree of relationship between 14-3-3η and Snail mRNA expression in FLS derived from patients and healthy individuals. The results showed that the expression of Snail was significantly correlated with 14-3-3η expression (*r* = 0.45, *p* < 0.001) (Figure 3A). We next investigated whether 14-3-3η regulates Snail expression. For this purpose, H FLS were stimulated with rh14-3-3η. We found an increase in 14-3-3η, as expected [4], and Snail mRNA expression in rh14-3-3η treated H FLS. However, rh14-3-3η failed to induce the mRNA expression of Slug, a member of the Snail family known to be differentially expressed and regulated [42] (Figure 3B). The results from Western blot analysis showed a similar effect of rh14-3-3η stimulation on Snail and 14-3-3η protein expression (Figure 3C). To further investigate the association between 14-3-3η and Snail expression, we evaluated the impact of 14-3-3η knockdown on the expression of Snail, and that of PTEN, which is a direct target of Snail repression activity [43]. We observed a significant decrease in Snail protein expression with a concomitant increase in PTEN (Figure 3D,E). Similar results were obtained at the mRNA level (Figure 3F). Collectively, these findings demonstrate that 14-3-3η regulates Snail expression and, presumably, transcriptional activity in RA FLS, events that could explain its ability to increase invadosome formation.

### 2.4. 14-3-3η Regulates Snail Expression and Invadosome Formation through Nuclear Exclusion of FOXO3

**Figure 4:**
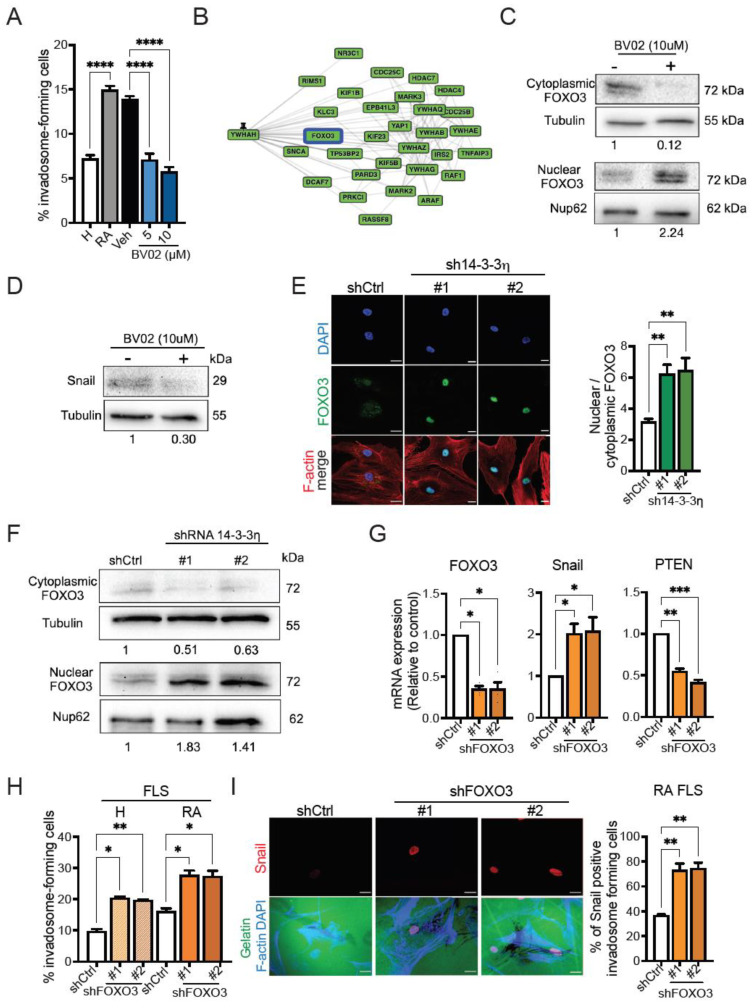
The effect of 14-3-3η-FOXO3 interaction on invadosome formation and Snail expression. (**A**) H FLS or RA FLS were cultured on Oregon green^488^-conjugated gelatin coverslips for 48 h and treated with BV02 at the indicated concentrations for 48 h. The percentage of invadosome-forming cells was calculated (*n* = 4). (**B**) Protein–protein interactions of 14-3-3η (*YWHAH*). In silico analysis was carried out using the GPS-Prot web-based database (http://gpsprot.org/, accessed on 17 March 2021); a minimum confidence score of 0.86 was chosen to investigate the interactions. The box highlighted in blue represents the 14-3-3η-binding protein of interest. (**C**,**D**) RA FLS were treated with 10 uM of BV02 or DMSO (control) for 48 h, and Western blot analysis was performed on the nuclear, cytoplasmic, or whole-cell extracts (*n* = 2–3). (**E**–**G**) RA FLS were transduced with non-targeting control (shctrl) or one of two 14-3-3η targeting shRNAs (#1 or #2). (**E**) Immunofluorescence analysis of FOXO3 localization. Representative confocal microscopy images showing the nucleus (DAPI, blue), FOXO3 (green), and F-actin (red). The associated graph shows the ratio of the mean fluorescence intensity of nuclear FOXO3 to that of cytoplasmic FOXO3. Data are expressed as the mean ± SEM of 12 microscopic fields in different areas of the slide per sample. Four independent experiments were performed. Scale bar = 20 µm. (**G**) Western blot analysis of the expression of FOXO3 in the nuclear and cytoplasm fraction. (**H**,**I**) H FLS or RA FLS were transduced with non-targeting control (shctrl) or one of two FOXO3-targeting shRNAs (#1 or #2). (**H**) qPCR analysis of FOXO3, Snail, and PTEN in RA FLS knocked down for FOXO3 (*n* = 3–5). (**I**) Transduced cells (RA FLS) were cultured on Oregon green^488^-conjugated gelatin coverslips, and the percentage of invadosome-positive cells was calculated. Immunofluorescence analysis of Snail nuclear localization in invadosome-forming cells. Representative confocal microscopy images showing the nucleus (DAPI, blue), F-actin (blue), gelatin (green), and Snail (red) (*n* = 3). The associated graph shows the percentage of Snail-positive invadosome-forming cells. Scale bar = 20 µm. Data represent the means ± SEM. * *p* < 0.05, ** *p* < 0.01, *** *p* < 0.001, and **** *p* < 0.0001, one-way ANOVA.

14-3-3 proteins are known to bind to more than 200 target proteins, including signaling molecules, enzymes, and transcription factors [44]. To gain insight into the mechanism by which 14-3-3η regulates Snail expression and invadosome formation in RA FLS, we initially tested the effect of inhibitors of 14-3-3 protein–protein interactions on invadosome formation. For this, we used BV02 and R18, two known inhibitors of docking sites for 14-3-3 proteins [45,46]. BV02 and R18 treatment led to a significant concentration-dependent decrease in invadosome formation in RA FLS (Figure 4A and Figure S1), suggesting the participation of 14-3-3 binding proteins. We next used the GPS-Prot database, a protein–protein interaction visualization platform, to predict 14-3-3η binding partners that are potentially implicated in invadosome formation. As expected, 14-3-3η (*YWHAH*) binds to several partners, including proteins involved in the cell cycle, apoptosis, and signaling pathways, such as the MAPK-Erk and TNFR1 pathways, as well as transcriptional regulators (Figure 4B). Among these, the transcription factor FOXO3 is of particular interest since it has been shown that the association between 14-3-3 proteins and FOXO3 triggers the nuclear export of FOXO3, leading to the inhibition of its tumor suppressor functions, such as the repression of EMT mediators [47,48,49]. In addition, down-regulation of FOXO3 has been shown to promote the expression of Snail and Twist-1 in several cancer cell lines [48,50,51].

To determine whether 14-3-3η participates in Snail upregulation and invadosome formation in RA FLS by inducing nuclear exclusion of FOXO3, we evaluated the effect of BV02 on FOXO3 subcellular localization by Western blot analysis. An increase in FOXO3 localization to the nucleus was found in BV02-treated RA FLS when compared with untreated cells (Figure 4C). Interestingly, this increase in FOXO3 nuclear localization was accompanied by a reduction in Snail expression (Figure 4D). To further support these findings, we evaluated the effect of 14-3-3η knockdown on FOXO3 localization by immunofluorescence and Western blot. Similar to the results of BV02 treatment, knockdown of 14-3-3η increased the nuclear localization of FOXO3 in RA FLS (Figure 4E,F). Next, to determine whether FOXO3 plays a role in invadosome formation via Snail repression, H or RA FLS were transduced using shRNAs that target two independent regions of FOXO3 mRNA. The results showed that FOXO3 knockdown significantly increased Snail expression while decreasing PTEN expression (Figure 4G). FOXO3 knockdown also caused a significant increase in invadosome formation by H FLS and RA FLS (Figure 4H).

Finally, to gain further insight into the role of the FOXO3–Snail axis in invadosome formation, we combined invadosome assays and immunofluorescence staining and evaluated the percentage of Snail-positive invadosome-forming cells in control and FOXO3-depleted RA FLS. The results showed that the percentage of Snail-positive and invadosome-forming cells was twofold higher in the FOXO3 knockdown condition than in control RA FLS (Figure 4I). 

Collectively, these results suggest that the 14-3-3η–FOXO3–Snail axis is involved in the formation of matrix-degrading invadosomal structures by RA-FLS and that nuclear exclusion of FOXO3 by 14-3-3η is one of the mechanisms involved.

## 3. Discussion

The progressive destruction of synovial-lined joints is a hallmark of RA, in which FLS are recognized as the main effectors of the degradation process. These cells have strong invasive properties and produce invadosomes that were previously shown to contribute to cartilage destruction [17,20,52]. In this study, we identified 14-3-3η as a novel regulator of invadosome formation and matrix degradation by RA synovial cells. Our data reveal that 14-3-3η exerts its function by promoting FOXO3 nuclear exclusion, resulting in increased Snail expression and subsequent invadosome formation. These findings expand our understanding of the pathological role of 14-3-3η in RA by revealing a potential mechanism by which 14-3-3η participates in cartilage destruction, i.e., through its ability to increase invadosome formation.

14-3-3η is one of seven members of the 14-3-3 family that are preferentially expressed at higher levels in certain pathological conditions [53,54,55]. In RA, high expression of 14-3-3η was previously associated with increased levels of ECM-degrading metalloproteinases and joint destruction [4]. Additionally, and in line with our findings indicating enhanced expression of 14-3-3η in RA synovial tissues and derived FLS cultures, a recent study indicated that 14-3-3η is highly expressed in macrophages and FLS from synovial tissues of RA patients [56]. However, a key issue remains as to how this molecule could be involved in joint destruction. In this study, we provide evidence supporting the implication of 14-3-3η as a main effector of ECM degradation. A strong positive correlation was observed between 14-3-3η mRNA levels and invadosome formation in primary FLS cultures. Moreover, depletion of 14-3-3η or inhibition of its intracellular scaffolding function reduced invadosome formation in RA FLS, whereas addition of 14-3-3η in FLS from healthy individuals had the opposite effect. These results suggest that in addition to the intracellular role of 14-3-3η described here, extracellular 14-3-3η may also be involved in invadosome-induced ECM breakdown. Extracellular 14-3-3s are known to induce tissue remodeling by binding to the cell-surface receptor aminopeptidase N (CD13), a receptor known to be expressed in RA FLS [57,58], suggesting the potential involvement of this receptor in invadosome formation—a possibility that will require further investigation.

As FOXO proteins are important effectors of intracellular 14-3-3 functions, we evaluated the expression of FOXO protein members in FLS. We showed that among the FOXO family members potentially expressed in the joint (FOXO1, 3, and 4) [33,35], FOXO3 was the most expressed in FLS from healthy or RA patients, but without significant difference between the two groups (Figure S2). This is consistent with previous studies that found no significant change in total FOXO3 expression in the sublining and lining layers of OA and RA synovial tissues [59,60]. Of note, a subsequent study revealed a strong increase in the phosphorylation pattern of FOXO3 in synovial membrane FLS from RA patients as compared to those from OA patients [35], suggesting that despite the lack of regulation of its expression, FOXO3 activity might be regulated in these FLS.

Modulation of subcellular localization of FOXO family members is a main event in the regulation of their transcriptional activity, and 14-3-3 proteins are known to be negative regulators of the function of FOXO proteins as they bind to the phosphorylated form of FOXOs, causing their translocation from the nucleus to the cytoplasm [35,47,60,61]. Such relocalization of FOXO proteins has been associated with aggressive behavior of cancer cells, as 14-3-3ζ-induced nuclear exclusion of FOXO3 resulted in increased proliferation and migration of tongue squamous cell carcinoma cells [62]. We therefore sought to determine whether a similar event could be associated with the pathological behavior of RA FLS. Immunofluorescence results indicate that a similar nuclear exclusion of FOXO3 occurs in RA FLS (Figure S3), and pharmacological inhibition of 14-3-3 protein–protein interactions or 14-3-3η depletion reversed the nuclear exclusion of FOXO3, resulting in reduced invadosome formation. These results highlight a role for 14-3-3η binding to FOXO3 in invadosome formation and suggest that this event may become an attractive target for countering the ECM-degradative capacity of RA synoviocytes. In keeping with this possibility, various compounds targeting 14-3-3 protein interactions are in development, with potential therapeutic implications in other disorders such as cancer and neurological disorders [63,64].

Our results further suggest that nuclear exclusion of FOXO3 is a mechanism by which 14-3-3η induces Snail expression and thus promotes invadosome formation. FOXO3 has been identified as a potent repressor of EMT-related transcription factors such as Snail, which has led to a better understanding of its tumor suppressive function [31,65,66,67]. In cancer cells, inactivation or knockdown of FOXO3 induces an EMT-like phenotype and thus promotes cell invasion and metastasis [48]. For example, loss of FOXO3 upregulates Snail and induces EMT in clear cell carcinoma [50], whereas in breast cancer cells, increased expression has the opposite effect [68]. Consistent with these studies, we observed that the knockdown of FOXO3, or the FOXO3 regulator 14-3-3η, significantly increased Snail expression and subsequent invadosome formation in H FLS and RA FLS. Furthermore, this knockdown enhanced the number of Snail-positive invadosome-forming cells, suggesting that Snail is a target of FOXO3 repressive activity. However, the question remains as to whether FOXO3 acts directly or indirectly to suppress Snail expression. Bioinformatic analysis using the Enhancer database revealed that the Snail promotor contains four consensus binding sites for FOXO3, raising the possibility that FOXO3 might directly regulate Snail expression through its ability to act as a transcriptional repressor. However, it has also been reported that FOXO3 can inhibit gene expression of Y-Box Binding protein-1 (YB-1), a protein that activates cap-independent translation of Snail mRNA [69], and/or induce mir-29, 30, and 34 transcription to reduce Snail mRNA levels [70,71,72], suggesting the potential for indirect post-transcriptional regulation. Therefore, the regulation of Snail expression by FOXO3 is complex, and further studies are required to determine the exact mechanisms involved in RA FLS.

Overall, our study identifies an intrinsic role for 14-3-3η in the control of the ECM-degrading ability of RA FLS that likely involves the down-modulation of FOXO3 repressive activity on Snail expression. These results provide new mechanistic insights into the regulation of joint degradation by RA FLS, while also highlighting novel ways to control their aggressive behavior. Thus, targeting the pathological role of FLS by preventing nuclear exclusion of FOXO3 by 14-3-3η could serve as a promising strategy to prevent joint destruction in RA patients.

## 4. Materials and Methods

### 4.1. Reagents

Mission lentiviral shRNAs targeting 14-3-3η (TRCN00000078163, TRCN00000369692), FOXO3 (TRCN0000235490, TRCN0000040099), or a scramble control sequence (SHC002), were obtained from Sigma Aldrich (St. Louis, MO, USA). Anti-14-3-3η and human recombinant 14-3-3η (rh14-3-3η) protein were kindly provided by Augurex (Vancouver, BC, Canada). Anti-Snail was obtained from Abcam (Cambridge, UK), anti-PTEN was obtained from Cell Signaling (Danvers, MA, USA), anti-FOXO3 was obtained from Santa Cruz Biotechnology (Dallas, TX, USA), and anti-α-tubulin was obtained from Sigma-Aldrich (St. Louis, MO, USA). Texas Red phalloidin, DAPI (4′,6-diamidino-2-phenylindole), and all secondary fluorophore-coupled antibodies were obtained from Invitrogen (Molecular Probes, Eugene, OR, USA). HRP-coupled secondary antibodies were obtained from Cell Signaling technology (Danvers, MA, USA).

### 4.2. Patients and Cell Culture

Human FLS were derived from synovial tissue collected during open surgery of patients diagnosed with RA or osteoarthritis (OA), or from healthy controls (H). Patients were recruited in collaboration with Dr. Frédéric Balg. The RA patients fulfilled the American College for Rheumatology (ACR)/European League Against Rheumatism (EULAR) criteria for RA classification [1]. FLS were isolated according to standard procedures [2] and were cultured in DMEM-F12 medium (Wisent, StBruno, Qc, Canada) supplemented with 10% FBS (Gibco BRL, Burlington, ON, Canada) and 40 mg/mL gentamycin (Wisent, St-Bruno, QC, Canada). Cells were used between passages 3 and 8 [3]. The study was approved by the Centre Hospitalier Universitaire de Sherbrooke Ethics committee, and written consent was obtained from all participants, protocol number 07-113.

### 4.3. Immunohistochemistry

Formalin-fixed, paraffin-embedded synovial tissue sections (5 µm) were deparaffinized and rehydrated. Immunohistochemical staining was performed according to the standard avidin–biotin immunoperoxidase complex technique [4], and the sections were incubated with anti-14-3-3η (Augurex, 1:100 in 2% BSA and 2% goat serum) or mouse isotype IgG (DAKO Agilent, Santa Clara, CA, USA,1:100). Diaminobenzidine was used as a substrate for the detection of the labeled proteins, and the sections were counterstained with Harris hematoxylin. Slides were scanned using a Hamamatsu Nanozoomer 2.0-RS scanner. For quantitative immunohistochemistry, six random fields (at original magnification 20X) for each patient were captured using NPD viewing software, and the intensity of labeling in the tissue sections was analyzed using the immunohistochemistry quantification technique as previously described [5]. The results are expressed as the sum of labeling intensity (density) relative to the total area.

### 4.4. RT qPCR

Total RNA was isolated using the TRIzol (Invitrogen, Carlsbad, CA, USA) extraction protocol, as previously described [6]. The RNA concentration was determined using a Nanodrop spectrophotometer, and 1 µg was reverse transcribed into complementary DNA (cDNA) using the QuantiTect reverse transcription kit (Qiagen, Mississauga, ON, Canada). Quantitative Real-Time PCR was performed on a Rotor-Gene 3000 (Corbett Research, Kirkland, QC, Canada) using a hot start SYBR Green qPCR master mix (BiMake, Houston, TX, USA). The cycling program was as follows: initial denaturation at 95 °C for 15 min, 35 amplification cycles with annealing T of 59 °C for 30 s, and final extension at 72 °C for 30 s. The results were quantified by the 2-ΔΔC(t) method, using the RPLP0 expression level for normalization. The primer pairs used are listed in Table 1. 

### 4.5. Plasmids and Transfections

pLKO.1-puro short hairpin RNAs targeting 14-3-3η, FOXO3, or control (scrambled) shRNA were obtained from Sigma-Aldrich. Viral particles were generated by transient transfection of 293T cells using the ViraPower lentiviral expression system (Invitrogen Thermo Fisher Scientific, Burlington, ON, Canada). For lentiviral transductions, cells were plated in 100 mm Petri dishes at a density of 3 × 10^5^ and infected with 1 mL of viral stock in 2 mL of optiMEM supplemented with 2 µL Polybrene (10 mg/mL; EMD Millipore, Etobicoke, ON, Canada). Transduced cells were selected by puromycin treatment for 72 h (2 µg/mL; Invivogen, San Diego, CA, USA).

### 4.6. Invadosome Assays

Coverslips were prepared with Oregon green^488^ conjugated gelatin (Invitrogen, Burlington, ON, Canada) at a final concentration of 0.5%, as previously described [7]. Thirty thousand cells were seeded onto each coverslip, incubated for 48 h, and fixed with 4% paraformaldehyde. Nuclei were stained with DAPI, and F-actin was stained with Texas Red phalloidin. Stained cells were visualized using a Zeiss Axioskop fluorescence microscope, and invadosomes were identified by F-actin-enriched areas of matrix degradation. Three fields of 100 cells (magnification 40×) were counted per coverslip to quantify the percentage of invadosome-forming cells.

### 4.7. Immunofluorescence and Microscopy

To measure the number of actin/cortactin-rich invadosomal structures, cells were seeded on 0.5% gelatin, incubated for 6 h, and stained with anti-cortactin antibody (1:50) and Texas Red phalloidin (1:200). Clusters of cortactin/actin were calculated for 20 cells per slide. For 14-3-3η localization at invadosome sites, RA FLS were cultured on Oregon green^488^ conjugated gelatin for 48 h and stained as described above using 14-3-3η antibody (1:100), Texas Red- or Fluor 350-conjugated phalloidin (1:200) and DAPI. Confocal images were acquired using a Fluoview 1000 scanning confocal microscope (Olympus, Richmond Hill, ON, Canada) in line with an inverted Olympus microscope equipped with a 60X oil immersion objective. Color channels were scanned sequentially to avoid overlapping signals. A set of z-stack images was collected at 0.25 μm intervals and reconstructed using FluoView software (Olympus). For FOXO3 localization, H or RA FLS were plated on coverslips overnight and fixed with 4% PFA for 10 min at room temperature. Cells were incubated with anti-FOXO3 (1:50), Goat anti-Mouse Alexa Fluor^488^ (1:200), Texas Red-conjugated phalloidin, and DAPI. Fluorescent images were obtained using confocal microscopy. The mean fluorescence intensity ratio of nuclear and cytoplasmic FOXO3 and the percentage of nuclear FOXO3 were determined and quantified using the Intensity Ratio Nuclei Cytoplasm Tool plugin of Image J (NHI, US) (Intensity Ratio Nuclei Cytoplasm Tool, RRID:SCR_018573). For Snail-positive invadosome-forming cells, invadosome assays were combined with immunofluorescence staining using an antibody directed against Snail (1:50), phalloidin, and DAPI. Three fields of 100 cells (magnification 40×) were counted per coverslip to quantify the percentage of Snail-positive invadosome-forming cells. Representative images were acquired using a Fluoview 1000 scanning confocal microscope (Olympus, Richmond Hill, ON, Canada).

### 4.8. Western Blotting

FLS were lysed with RIPA buffer supplemented with phosphatase inhibitor cocktail and protease inhibitor mix, and immunoblotting was performed as previously described [5]. Membranes were blocked with either 5% BSA or 5% non-fat dry milk and incubated overnight at 4 ℃ with the following antibodies: anti-14-3-3η (1:200), anti-Snail (1:100), anti-PTEN (1:100), anti-FOXO3 (1:100), anti-nup62 (1:1000), or anti-α-tubulin (1:1000). The secondary antibody was a peroxidase-conjugated anti-rabbit or anti-mouse antibody, depending on the source of primary antibody used. Immunoblots were revealed using the Cytiva Amersham^TM^ ECL Select^TM^ Western blotting detection reagent (Little Chanlfont, United Kingdom). For nuclear/cytoplasmic cell fractions, cells were trypsinized, collected, and washed twice with cold PBS. For cytoplasmic fractions, cells were resuspended in lysis buffer (10 mM Tris-Base pH7.4, 10 mM NaCl, 3 mM MgCl_2_, 0.5 mM EDTA, 0.5 mM EGTA, 0.5 mM DTT, and protease inhibitors) for 15 min on ice, followed by the addition of NP40 buffer (lysis buffer supplemented with 0.2% NP40); they were then centrifuged, and the supernatant was collected. For nuclear fractions, the cell pellets were resuspended in nuclear extraction buffer (10 mM Tris-Base pH7.3, 400 mM NaCl, 5 mM EGTA, 30 mM MgCl_2_, 1 mM DTT, antiprotease cocktail, and 10% glycerol) for 20 min; they were then centrifuged, and the supernatants were collected. The fractions were analyzed by Western blotting. Band intensities were analyzed using Image Lab^TM^ software (Version 6.0.0, Bio-Rad Laboratories, Mississauga, ON, Canada).

### 4.9. Protein–Protein Interaction

The GPS-Prot database was used to explore the protein–protein interaction network of 14-3-3η (gene name: *YWHAH*). The 14-3-3η interaction network was visualized on the web-based platform. To select the top 14-3-3η interactors, a minimum confidence score of 0.86 was used.

### 4.10. Statistical Analysis

The data were analyzed using Graphpad software. Unpaired Student’s t test or one-way ANOVA was used to assess statistical significance. *p* values of <0.05 were considered significant.

## Figures and Tables

**Table 1:** qPCR primer pairs.

Gene	Forward Primer (5′→3′)	Reverse Primer (5′→3′)
*RPLP0*	GATTACACCTTCCCACTTGC	CCAAATCCCATATCCTCGTCCG
*Snail*	CCTTCGTCCTCCTCCTCTACTT	TTCGAGCCTGGAGATCCTT
*14-3-3η*	CTATGAAGGCGGTGACAGAGC	CCTTGTAGGCAGCTTCAGAAG
*FOXO3*	GACCCTCAAACTGACACAAGA	TGGCGTGGGATTCACAAA
*PTEN*	CCCACCACAGCTAGAACTTATC	TCGTCCCTTTCCAGCTTTAC

## Data Availability

No new data were created or analyzed. Data sharing is not applicable to this study.

## Supplementary Materials

The following are available online at https://www.mdpi.com/article/10.3390/ijms23010123/s1.
