# Supplementary material for: 14-3-3η Promotes Invadosome Formation via the FOXO3–Snail Axis in Rheumatoid Arthritis Fibroblast-like Synoviocytes
Source: Int J Mol Sci. 2021 Dec 23;23(1):123. doi: 10.3390/ijms23010123 (PMC8745703; doi:10.3390/ijms23010123)
Supplement: Supplementary file 1 [file ijms-23-00123-s001.zip › ijms-1513945-supplementary.pdf]

Supplementary Figures

14-3-3η Promotes Invadosome Formation Via the FOXO3-Snail Axis in Rheumatoid Arthritis Fibroblast-Like Synoviocytes

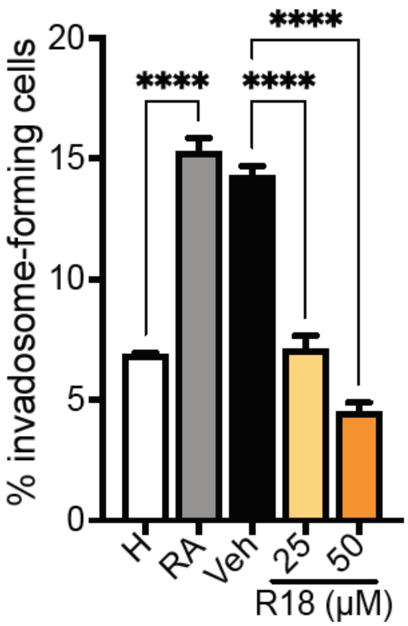

Figure S1. Effect of the 14-3-3 inhibitor R18 on invadosome formation. H FLS or RA FLS were cultured on Oregon green[488] conjugated gelatin coverslips for 48 hours, treated with R18 at the indicated concentrations for 48 hours and the percentage of invadosome-forming cells was calculated. Data represents the means ± SEM n ≥ 3. **** $p < 0.0001$, one-way ANOVA.

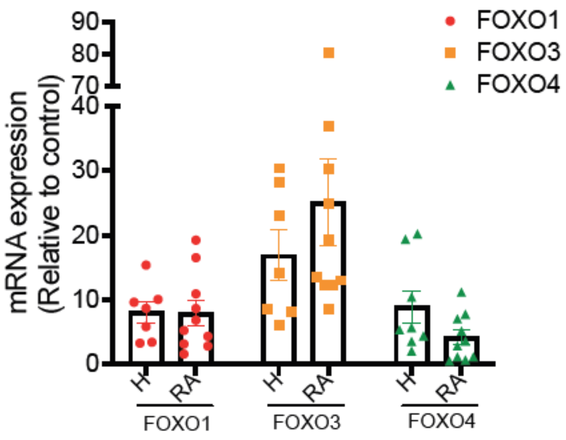

Figure S2. FOXOs expression in H and RA FLS.

RT-qPCR analysis of FOXO1, FOXO3 and FOXO4 mRNA expression in H FLS and RA FLS was performed using RPLP0 as a housekeeping gene. Results are expressed as relative mRNA expression to RPLP0 (ΔCt). Data represents the means ± SEM n ≥ 11, unpaired t-test.

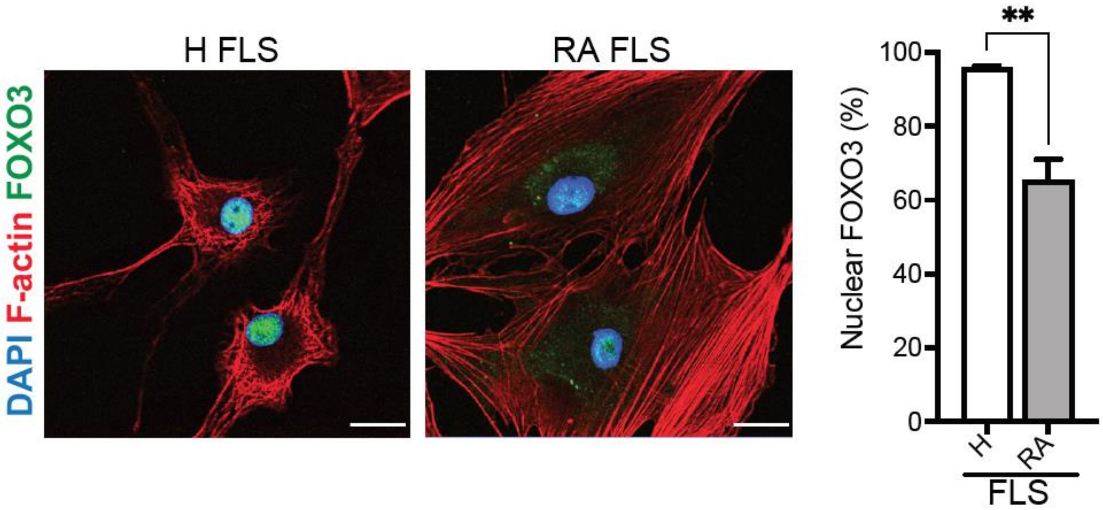

Figure S3. FOXO3 localization in H FLS and RA FLS.
Immunofluorescence analysis of FOXO3 nuclear localisation in H FLS and RA FLS. Representative confocal microscopy images showing nuclei (DAPI; blue), F-actin (phalloidin; red) and FOXO3 (green). The associated graph shows the percentage of nuclear FOXO3. Data are expressed as the means ± SEM of 12 microscopic fields from different areas of the slide per sample (H FLS (n=3), RA FLS (n=3)). Scale bar=20µm. ** $p < 0.01$, unpaired t-test.
